# Awareness of endocrine-disrupting chemicals among medical students and physicians: a cross-sectional study

**DOI:** 10.1080/10872981.2025.2585645

**Published:** 2025-11-19

**Authors:** Gokcen Unal Kocabas, Su Ozgur, Niyazi Emre Kursunoglu, Isabel Raika Durusoy Onmus, Yigitcan Yuksel, Banu Sarer Yurekli

**Affiliations:** a Ege University School of Medicine, Department of Endocrinology and Metabolism, Izmir,Turkey; b Ege University Translational Pulmonary Research Center-EgeSAM, Izmir, Turkey; c Regional Hub for Cancer Registration in Northern Africa, Central and Western Asia, WHO/IARC GICR, Izmir, Turkey; d Ege University School of Medicine, Department of Pediatrics, Izmir, Turkey; e Ege University School of Medicine, Department of Public Health, Izmir, Turkey; f Dokuz Eylul University School of Medicine, Izmir, Turkey

**Keywords:** Environmental health, endocrine disruptors, medical curriculum, awareness, student

## Abstract

Endocrine-disrupting chemicals (EDCs) have diverse sources of exposure in everyday life. Raising public awareness is crucial, considering the undeniable role of individual choices in EDC exposure. Given the critical role of physicians in public health education and the increasing importance of preventive strategies, it is essential to evaluate EDC awareness among both current and future healthcare providers. This study aimed to assess the level of EDC awareness among Turkish medical students and physicians using a validated scale, and to examine how this awareness relates to individuals' general attitudes toward preventive health, as measured by the Healthy Life Awareness (HLA) Scale. This cross-sectional study employed the endocrine Disruptor Awareness Scale (EDCA) and the Healthy Life Awareness Scale (HLA) to assess participants' knowledge and attitudes. The survey was disseminated electronically through e-mail. We reached a total of 617 participants. Three hundred eighty-one were medical students, and 236 were physicians. The median EDC general awareness score was significantly higher in physicians compared to students (2.12[1.5] vs 2.87[1.63], *p* < 0.001). The mean EDC awareness total score was also higher in physicians (3.4 ± 0.54 vs 3.63 ± 0.6, *p* < 0.001). Female physicians' awareness was significantly higher than their male counterparts (3[1.38] vs 2.75[1.56], *p* = 0.027). Age and healthy life awareness scores significantly correlated with EDC awareness scores. In particular, endocrinologists' scores were significantly higher than other subspecialties (total score 3.59 ± 0.58 vs. 3.96 ± 0.56, *p* = 0.003). The findings reveal a significant gap in EDC awareness among medical students, highlighting a lack of sufficient curricular coverage at the undergraduate level. The positive associations observed between EDC awareness, age, and healthy life awareness suggest that individual health consciousness and postgraduate experience contribute to greater awareness. These results underscore the importance of incorporating environmental health, particularly the endocrine disruptors, into medical curricula at various stages of training.

## Introduction

Endocrine-disrupting chemicals are exogenous agents that interact with the synthesis, secretion, transportation, metabolism, binding, or elimination of hormones naturally found in the body [1]. Endocrine-disrupting chemicals can partially or entirely mimic hormones and show agonist effects, which means they can activate hormone receptors, or antagonist effects, which means they can block the action of hormones.

They can be industrial solvents, plastics or plasticizers, pesticides, drugs, and natural chemicals [2]. Apart from being small molecules, their structures may differ significantly. Their sources are also very diverse. They can enter the food chain from soil, water, and contaminated animals. Although people who work with pesticides, fungicides, and industrial chemicals, in particular, have a higher risk of exposure, this is a widespread public health problem, as there is a vast area of contamination, ranging from household items and cosmetics to consumed foods.

Age at the time of exposure is crucial. Exposure from the intrauterine period has been reported, and the potential for future disease increases with effects on the developing organism. Some endocrine-disrupting chemicals (EDCs) have very long half-lives, meaning they can persist in the environment and in our bodies for extended periods, potentially even from one generation to the next. Others have short half-lives, meaning they break down more quickly. The fact that they can cause diseases years after exposure and are effective even at low doses makes the problem even more difficult.

The increasing incidence of breast, prostate, and testicular cancers, diabetes, obesity, and decreased fertility in the last 50 years has also been associated with endocrine disruptors [3]. In addition, an association has been found with early puberty in women, polycystic ovary syndrome, and premature ovarian failure. Decreased sperm quality and decreased fertility have also been shown in men.

The Endocrine Society, a prominent international organization in the field of endocrinology, drew attention to the issue by publishing two position papers in 2009 [1] and 2015 [4]. The European Office of the World Health Organization, including Turkey, has published a declaration to determine the risks of exposure to endocrine disruptors at the country level [2]. This declaration recommends identifying institutions that will address these issues, coordinating activities on EDC at the national level, establishing an EDC policy that determines priorities, participating in information exchange networks, and raising awareness. The activities regarding endocrine-disrupting chemicals are relatively new to our country. The Society of Endocrinology and Metabolism of Turkey recently published a position paper about EDCs [5]. The Ministry of Health recently declared a position statement about the health effects of endocrine disruptors [6].

Public health primarily focuses on preventing diseases at the population level, while medicine is concerned with the prevention, diagnosis, and treatment of illnesses in individual patients. Preventive medicine, widely regarded as one of the most cost-effective healthcare strategies, is increasingly recognized as essential for managing and reducing the escalating costs associated with treating chronic diseases. EDC exposure is influenced by personal behaviors, corporate decisions, and national laws. While the development of regulatory policies is important, their implementation may take considerable time. Personal behaviors, such as dietary habits and the use of personal care products, have a significant impact on an individual's exposure to endocrine-disrupting chemicals.

Environmental risk assessment remains a rarely addressed component of clinical encounters worldwide, including patient education practices [7]. A qualitative study among environmental clinicians concluded that educational resources are lacking in environmental health [8].

In two studies conducted among pregnant women [9,10], healthcare professionals were considered reliable sources of information; however, they were infrequently consulted. Instead, participants more often relied on easily accessible sources such as media and self-help books, which were perceived as less reliable but still valuable by some. Although endocrine disruptors are increasingly recognized as a public health concern, the extent of awareness among healthcare providers remains unclear. Particularly, there is a paucity of data regarding medical students’ understanding of this issue, despite their future role in health communication. Therefore, this study aimed to assess the awareness of endocrine-disrupting chemicals among Turkish medical students and physicians using a validated scale, and to investigate its association with general healthy life awareness.

## Materials and methods

### Study design and setting

This was a cross-sectional, questionnaire-based study conducted between March 2024 and December 2024 at Ege University School of Medicine, Izmir, Turkey. All participants provided informed consent digitally before accessing the questionnaire. The study protocol was approved by the Ege University Ethics Committee (approval number 23-8T/3) and conducted in accordance with the Declaration of Helsinki.

### Participant recruitment

A survey form was constructed, including demographic information (age, gender, educational status, and specialty status, if applicable), the Healthy Life Awareness Scale, and the Endocrine Disruptor Chemicals Awareness Scale. Participants were reached via institutional email directories and professional contact networks, including hospital departments and student networks in medical schools. The use of institutional channels ensured the authenticity of respondents as affiliated medical students or physicians. Duplicate responses were limited by unique email validation. Participation was voluntary and anonymous. Participants who failed to complete the entire survey or provided inconsistent demographic data were excluded from the final analysis.

### Scales

The Endocrine Disruptor Awareness scale (EDCA) is a validated instrument developed by Tan et al. [11]. consisting of 24 items with a 1−5 Likert-type scoring system. It includes three subcategories: general awareness, impact, and exposure and protection. The mean points of the scale subgroups and total were interpreted as follows: 1−1.8: very low; 1.81−2.6: low; 2.61−3.4: moderate; 3.41−4.2: High; 4.21−5:very high. The categorization was performed in accordance with the original scale developers' classification to enhance interpretability and facilitate comparison with future applications of the same scale.

We also used the Healthy Life Awareness Scale (HLA) to determine the general healthy life preferences of the participants. The scale was developed and validated by Ozer et al. in the Turkish population [11]. The Healthy Life Awareness scale consists of 15 items with 5-category Likert-type scoring scale from 1 to 5 grouped into four subdomains: change (items 1–5), socialization (items 6–9), responsibility (items 10–12), and nutrition (items 13–15). The higher score indicates a higher level of healthy life awareness.

### Sample size

The study is based on the fundamental hypotheses and includes individuals aged 18−75 who are either medical school students or medical school graduates. The sample size for the study was calculated based on the assumption that the population is unknown.

A review of the literature revealed that only a limited number of studies have assessed awareness of endocrine-disrupting chemicals among healthcare professionals, and none have provided prevalence estimates that could directly guide sample size determination. Therefore, the prevalence was assumed to be *p* = 0.50. Accordingly, within a 95% confidence interval, with a margin of error of 6% and a prevalence rate of *p* = 0.50 in the population, the minimum required sample size was calculated to be 267 individuals. Considering potential losses (e.g., missing or incomplete data), an additional 10% of participants were planned to be included. Consequently, the minimum number of participants required for the study was calculated to be 294 individuals, comprising 200 students and 94 graduates [12].

### Statistical analysis

All statistical analyzes were performed using IBM SPSS version 25.0 (Chicago, IL, USA). Descriptive statistics were reported as mean ± standard deviation (SD) if normally distributed and median [interquartile range-IQR] if non-normally distributed for continuous variables, while frequencies and percentages were provided for categorical variables. Incomplete responses were excluded listwise. No imputation was performed for missing values. The normality assumption of continuous variables was assessed and based on this, appropriate parametric or non-parametric tests were applied Since most continuous variables were not normally distributed, non-parametric tests were primarily used: the Mann–Whitney U test for two-group comparisons, the Kruskal–Wallis test for comparisons across more than two groups, and Spearman’s rank correlation for associations. Parametric alternatives (Student’s t-test, one-way ANOVA, Pearson’s correlation) were considered only when distributional assumptions were met. Linear regression analysis was used to investigate the relationship between variables. A final model was constructed using a backward stepwise method, including all variables. A *p*-value of less than 0.05 was considered statistically significant.

## Results

A total of 617 medical students and physicians were included in the final analysis. 381 were medical students, and 236 were physicians. Median healthy life awareness scale subgroups of change, socialization, and responsibility were significantly higher in physicians compared to the students, while the nutrition subgroup and total score were not significantly different (Table 1).

**Table 1.** Healthy life awareness and endocrine-disrupting chemicals awareness (EDCA) scores of students and physicians *mean ± SD.

	Students(*n* = 381)	Physicians (*n* = 236)	*p* value
Age	22(3)	43(16)	<0.001
Gender(F/M)	231/149 (60.8%-39.2%)	147/90 (62%-38%)	0.759
HLA-Change	21(4)	22(4)	0.001
HLA-socialization	14(4)	13(5.75)	<0.001
HLA-Responsibility	12(4)	12(3)	<0.001
HLA nutrition	11(4)	11(4)	0.423
HLA total	58(12)	58(9)	0.599
EDCA-General awareness	2.12(1.5)	2.87(1.63)	<0.001
EDCA-Impact	4.12(0.88)	4.12(0.88)	0.399
EDCA exposure-protection	4(1)	4(1)	0.971
EDCA total*	3.40 ± 0.54	3.63 ± 0.6	<0.001

Endocrine disruptor awareness scores, general awareness subscale, and the total score were significantly higher in the physician group. The EDCA impact and exposure-protection subscales did not show significant differences between the physicians and the students. (Table 1).

When the EDCA total score of the first three grades of medical school (preclinical phase) was compared to that of students in the 4th grade and higher (clinical phase), there was no significant difference (*p* = 0.053). The clinical phase students' awareness score was significantly lower compared to the physicians (3.44 ± 0.71 vs 3.63 ± 0.81, *p* < 0.001).

**Figure 1.**
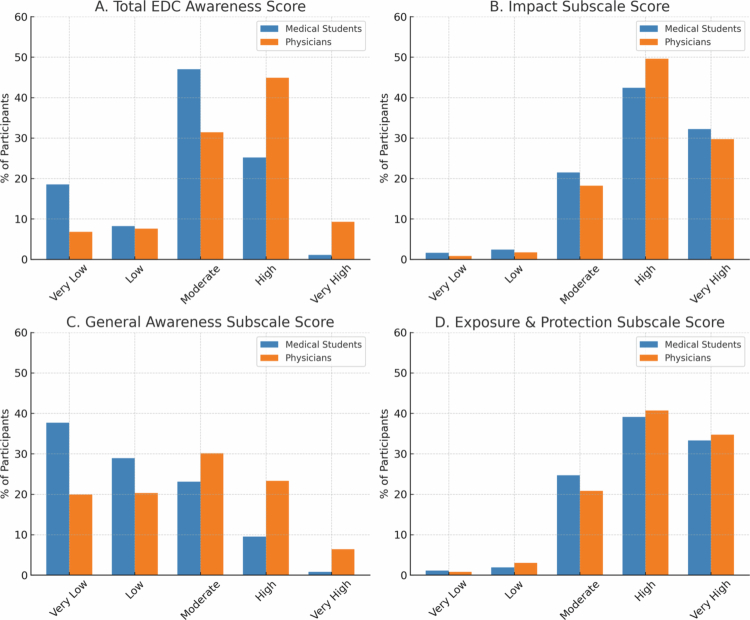
Awareness levels of medical students and physicians across EDC awareness subscales. A. Total EDC awareness score B. Impact subscale score C. General Awareness subscale score D. Exposure & Protection subscale score.

**Figure 2.**
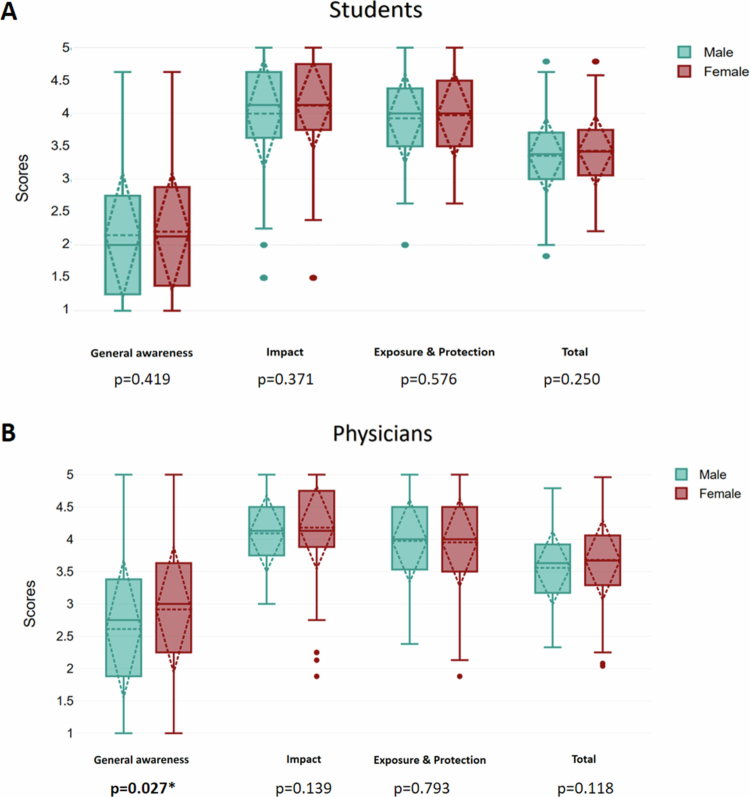
EDC awareness scores of students (Figure 2A) and physicians (Figure 2B) according to gender.

The majority of medical students (47.6%) scored moderate in the total EDCA score, while 49.8% of the physicians scored high (*p* < 0.001) (Figure 1A). In terms of the general awareness subscale, more than half of the students scored low or very low, while 39.3% of the physicians scored low/very low, and 30.8% of the physicians scored moderate (*p* < 0.001) (Figure 1C) There was no significant difference of awareness levels in impact and exposure-protection subscales between students and physicians (Figure 1B and 2D).

When the endocrinology subspecialists were compared to the other physicians, the general awareness score was high or very high in **61.5%** of the endocrinologists, compared to 26.1% of the remaining physicians (*p* < 0.001). The endocrinologists also scored higher than the remaining physicians in EDCA total score (the ratio of high/very high scores 80.7% vs 58.8% *p* = 0.007). EDC impact subscale (**92.3%** vs. 80,1% (*p* = 0.35) and exposure-protection subscale **88.4%** vs. 74.7% (*p* = 0.145) were not statistically significant.

Working in primary, secondary, or tertiary healthcare centers or private practice was not statistically significant for EDC awareness scores (data not shown).

The general awareness score of female participants was significantly higher in the physician group (3 [1.38] for females vs 2.75 [1.56]) for males; *p* = 0.027). The total EDC awareness score, EDCA impact, or exposure-protection subscales did not show significant differences in terms of gender for either the student or physician groups. (Figure 2A, 2B).

Similarly, the HLA scores of females were also significantly higher (57.5 [7] for males vs 59 [11] for females, *p* = 0.048).

The length of service (the time after graduation) in years was positively correlated with EDC awareness (Spearman's Rho 0.245, *p* < 0.001).

The EDC awareness total score was also positively correlated with age (Spearman's Rho, 0.228; *p* < 0.001) and healthy life awareness score (Spearman's Rho, 0.287; *p* < 0.001) in the physician group. There was no significant correlation between HLA score and age or length of service.

To identify the predictors of EDC awareness, a backward linear regression analysis was performed using age, gender, group status (students vs physicians), and healthy life awareness score as independent variables. Confounders were selected based on prior literature and clinical relevance. The final model retained age and healthy life awareness after stepwise elimination as significant predictors. The model was statistically significant with an adjusted R² of 0.126 (F(2.614) = 45.33, *p* < 0.001). Regression coefficients and confidence intervals are presented in Table 2.

**Table 2.** Linear regression analysis for predictors of Endocrine Disruptor Awareness (EDCA Total Score).

Predictor variable	B coefficient	95% Confidence interval	*p*-value
Age	−0.011	0.008—0.015	< 0.001
Healthy life awareness score	−0.018	0.013—0.024	< 0.001

Model summary: Adjusted R² = 0.126; F(2, 614) = 45.33; *p* < 0.001. Note: Gender and group status (student vs. physician) were excluded from the final model following backward elimination.

## Discussion

To our knowledge, this is the first study to demonstrate that medical students in Turkey have significantly lower awareness of endocrine-disrupting chemicals (EDCs) compared to physicians. While previous studies have assessed public and professional awareness of EDCs, research focusing specifically on physicians is limited, and to date, no studies have examined medical students.

Rouillon et al. conducted a survey of French pregnant women. They scored the knowledge using a questionnaire including definition, ability to give some examples, source, and way of exposure, and knowledge about the avoidance of EDC. Fifty-three percent of the women had never heard about EDCs. The mean knowledge score was 42.9 ± 9.8 out of 100 [9]. Similarly, a French local survey conducted on the general population also showed that 47.4% of the population had not heard about EDC [13]. In our study, even among medical students or physicians who are expected to be better informed due to their education in health sciences, awareness levels were alarmingly low.

Kelly et al. studied a focus group on public awareness and risk perception of EDCs, revealing that most participants were not aware of EDCs. The ones who were familiar had learned them during an undergraduate science degree, or were personally suffering from an endocrine disorder, or had seen social media posts. [14]. Our findings are consistent with this pattern, suggesting that medical students’ knowledge of EDCs likely stems from incidental sources rather than structured educational content.

In the aforementioned study, participants were aware of specific EDCs, namely pesticides, and bisphenol-A (BPA), but their knowledge of the chemicals was limited to recalling names [14]. The scale we used did not include specific EDC names, except for pesticides. Despite being asked whether they were aware that pesticides have EDC effects; 65.3% of students and **81.9%** of the physicians agreed, but only 14.8% of students and 21.9% of physicians felt confident in giving examples of synthetic EDCs. This suggests a superficial familiarity lacking in scientific depth or practical application.

A survey conducted on public participants of the Belgian population revealed similar results, in which 48% of the population had never heard of endocrine disruptors. Approximately one-third of the respondents were aware of the harmful substances in pesticides, but the plastics in packaging material, care products, and toys are less well-known [15].In our study, 19% of students and 36.7% of physicians reported being able to provide examples of materials in which EDCs are used.

Sunyach et al. assessed environmental health knowledge among perinatal healthcare professionals in France; including midwives, nurses, and a smaller proportion of physicians. While the main focus of the study was environmental health, endocrine-disrupting chemicals were also covered as part of the questionnaire. Interestingly, 98% of participants were considered to have adequate awareness, attributed to the strong support from the media, public authorities, and national policies, as noted by the authors [16]. In contrast, awareness initiatives in Turkey remain limited. Only recently has the Ministry of Health issued a position paper on EDC-related health effects [6], and the topic is scarcely addressed by mainstream media or policymakers. They also noted that even among physicians, over 60% had no formal training in environmental health [16]. Similarly, an American survey found that only one in fifteen obstetricians had received such training [17]. In our study, we did not collect data on EDC training of the participants, but as far as we know, no medical school curriculum involves environmental health, in particular EDC.

Marquillier et al. found that many French prenatal care professionals lacked sufficient information about EDC risks during pregnancy [18]. In our study, 86% of students and 84% of physicians agreed that environmental history should be taken during pregnancy, and preventive advice should be offered. However, the high level of agreement may reflect socially desirable responding, as the item was likely perceived as expected or appropriate. Thus, although the result seems positive, it may not accurately indicate participants’ actual knowledge or preparedness to act.

Our results also showed a positive correlation between EDC awareness and age, consistent with the earlier findings of Marquillier et al. [18]. However, this trend is not universal. A Croatian study examining healthcare professionals’ knowledge about brominated flame retardants found no association between age or interest level and actual knowledge [19].

A systematic review by Pravednikov et al. [20]. classified the determinants of EDC risk perception into sociodemographic, family-related, cognitive, and psychosocial categories. Consistent with our findings, they reported that older individuals tend to be more concerned about EDCs. Prior studies have shown that women perceive greater risks associated with EDCs; a trend we also observed in our physician group, where female participants scored higher on the general awareness subscale.

Also, individuals with pre-existing medical conditions have been shown to express greater concern about EDC risks [20]. In our study, we utilized the Healthy Life Awareness scale to indirectly determine the individual health concerns of the participants. The positive correlation between HLA scores and EDC awareness among both students and physicians supports the idea that those more engaged in health issues are also more sensitive to environmental risk factors such as EDCs. In our regression model, HLA score remained a significant predictor of EDC awareness even after adjusting for age, gender, and education status. This implies that a general tendency toward proactive health behavior, rather than formal medical education alone, may drive awareness of EDCs. While personal health awareness may contribute to EDC knowledge, relying solely on individual motivation is insufficient to ensure a comprehensive understanding among future healthcare professionals. Structured training is needed to prepare medical students to recognize and address environmental health risks such as EDCs.

Although the number of subspecialties in our participant group was limited, we observed that endocrinologists had significantly higher general awareness scores compared to other physician groups. This finding aligns with the European Society of Endocrinology’s training recommendations, which explicitly include endocrine-disrupting chemicals as a core topic in subspecialty education [21]. EDC-related content is also commonly featured in postgraduate settings such as conferences, seminars, and academic lectures, which may contribute to the elevated awareness observed among endocrinologists.

Studies among non-medical university students in Malaysia reported low levels of EDC knowledge, with average scores ranging from 37% to 50% [22,23]. Although these studies did not specifically focus on medical students, the findings highlight a general lack of awareness at the university level.

In a U.S.-based curriculum intervention, the inclusion of environmental health topics in medical education significantly improved students’ preparedness to discuss these issues with patients after one year [24]. This provides strong support for integrating EDC education into formal curricula to build sustained awareness and clinical competence among future healthcare providers.

One of the strengths of our study is the use of a valid and reliable scale specifically developed to assess EDC awareness among healthcare professionals. Unlike previous studies that employed general surveys or semi-structured interviews, our tool was tailored to the medical context and targeted both students and practicing physicians.

However, our study also has several limitations. First, despite efforts to include participants from different regions and faculties, the majority of respondents were based in Izmir, limiting the generalizability of our findings to the broader national population. Second, the small number of physicians with subspecialties prevented us from drawing robust conclusions regarding EDC awareness across different medical disciplines. Although the survey was open to all eligible participants through institutional email and professional networks, the voluntary nature of participation may have led to selection bias, potentially favoring individuals with a greater interest in environmental or health-related topics. However, the use of institutional mailing lists and professional channels likely increased the likelihood that respondents were genuinely affiliated and relevant to the target population.

In conclusion, to the best of our knowledge, this study is the first to evaluate EDC awareness among both medical students and physicians using a validated measurement tool in the literature. Our findings suggest that current awareness levels are insufficient and that educational reform is urgently needed. Future research should involve larger, nationally representative samples encompassing a broader range of medical specialties to inform targeted interventions and curriculum development. In light of the identified knowledge gap, we propose the structured integration of endocrine-disrupting chemicals (EDCs) into undergraduate and postgraduate medical curricula. EDC-related content may be embedded within existing modules such as physiology, endocrinology, public health, or environmental medicine. Additionally, case-based discussions and interdisciplinary seminars focused on environmental health could reinforce practical understanding**.** For practicing physicians, continuing medical education (CME) initiatives may serve as an effective means to bridge existing gaps in EDC-related knowledge. Positioning EDCs within core learning outcomes would not only ensure consistent exposure across institutions but also equip future physicians with the necessary competencies to address environmentally mediated health risks in clinical practice.

## Ethical approval and consent to participate

Written permissions were obtained for the use of both scales. This study was approved by Ege University local ethics committee with approval number 23-8T/3. All participants included in the study provided informed consent. This study was performed in adherence with the Declaration of Helsinki.

## Consent for publication

Not applicable.

## Data Availability

The datasets used and/or analyzed during the current study are available from the corresponding author on a reasonable request.

## Abbreviations

EDC: Endocrine-disrupting Chemicals
EDCA: Endocrine-disrupting chemicals awareness
HLA: healthy life awareness
IQR: interquartile range
SD: standard deviation
BPA: bisphenol A
